# Address

**Published:** 1880-07

**Authors:** J. S. Cassidy


### ADDRESS.

BY J. S. CASSIDY.

*Gentlemen of the Kentucky Dental Association:*

During the last past twelve months our profession has kept pace with the very highest and best examples of scientific attainment. Of this fact every one of you is well aware, and it is not too much of self-laudation for us to assert that the phenomenal progress of conservative dentistry has been developed and continued by just such associations as this of ours.

We meet and discuss methods and theories of practice, and although each member may advance ideas, which of themselves are not apparently of much significance, the collective truths produced thus are the jewels in the coronet of our young profession, whose brilliancy attracts the admiration of even the shallow skeptic.

Unstinted praise of those who constitute the active membership of any dental association, is necessary in order to properly appreciate the negative non-progressive qualities of many brethren who never partake of this feast of reason and flow of soul. In our own State, a very large majority of our profession is made up of the latter class. They are cordially and regularly invited to come, but, wrapped up in their invulnerable incrustation of selfishness, they do not come; one, because he may be too busy, and wise enough already, in his own conceit; another is afraid of personal contact with more ambitious, and perhaps brighter minds; and still another, alas, too poor in purse to permit of even so brief a holiday; and so the membership in our State Society continues comparatively small. All honor, therefore, to those who, having already received the highest honors it can confer, are seldom absent from its meetings. Perhaps I should not make allusion to any individual brother, as being most worthy of our gratitude and emulation, but I am sure that I do not make invidious distinction by expressing in words of sincerity and truth, my thanks to that noble Nestor of Kentucky Dentistry, Dr. Wm. H. Goddard, for the faithfulness of his devotion to our society, his wise counsel and never-flagging energy in assisting in the conduct of its government.

May time deal gently with him, and may the fruition of the brightest hopes of other days be repeated to him, in the fulness of many years yet to come.

In thinking over the recent evidences of progress in dentistry, it seemed to me that if we properly ignored the late introduction of all patent dental appliances and preparations, you would already be familiar with the legitimate phases of the progress made since our last meeting, and would therefore not require of me, the duty of making unnecessary mention of matters of this kind.

There is one line of experiment, however, which appears to be of sufficient interest to the profession at large to justify special mention, viz.: the effort to obtain reliable plastic materials for fillings. With the object of determining the value of the zinc phosphate cements lately introduced, I instituted some experiments looking to the properties of "resistance to chemical changes," "hardness," and "non-irritant action on exposed pulps." About six weeks ago, several teeth were filled with the cement prepared by Prof. Pierce, of Philadelphia, and also with a similar cement prepared by myself. They were allowed to remain dry over night, and were then placed in a variety of alkali, neutral and acid solutions. Such alkalies as saturated solutions of potassium carbonate have had no appreciable effect thus far, but a very dilute solution of aqua-ammonia removed every particle of a large filling in thirty minutes. A concentrated solution of sodium chloride has produced no appreciable change, although the tooth itself is considerably abraided. Dilute sulphuric acid disentegrates the filling rapidly and with greater ease, than either dilute nitric acid or hydrochloric acid. With fermenting saliva, enclosed in a small vial, the filling appears incompatable, inasmuch as its superficial substance is now easily removed by an excavator. In short, I am convinced that zinc phosphate is not much more reliable than the older preparations, in its resistance to chemical changes when exposed to the varying conditions present in the oral cavity.

Its superior hardness is obviously not of much value, unless protected from chemical action. It appears to bear the palm, however, over all other substances as a non-irritant covering to exposed pulps. This is merely an opinion based on about fifteen wellmarked cases of recent treatment, in my own practice; most of them being such as would have suggested a few years ago, the necessity of either devitalization or extraction. The remarkable tolerance manifested, in nearly every instance, by the pulp to the fluid cement, was at first considered a dangerous symptom, but a number of the pulps thus experimented with have been examined after a trial of several weeks, and found to be still possessed of normal vitality. In this respect at least, it is generally conceded that the phosphatic treatment accomplishes much more success than any previously known methods. It is also generally acknowledged that the usefulness of the phosphates as filling materials, is but one step in advance of the oxy-chloride of zinc, so long employed in similar cases. They cannot take the place of gold, nor even of amalgams; and indeed, it is the substitution of the latter class only, by something more acceptable, durable and sightly, that should engage our attention while searching for the ideal filling.

It is a matter for mutual congratulation that dental education has received an impetus during the last few years; and this always interesting subject now presents many questions pro and con, as to whether the numerous dental schools springing up at different points in our country, are a cause or a consequence of a demand for a higher degree of attainment than was or could be, conferred by the older institutions. If their greatly increased number should compel a corresponding increase in excellence on the part of all who enter our profession, then indeed, they are filling a want long felt; but if they are brought into existence simply as a consequence of the fact that by legal enactment, nearly all who now enter our profession must pass through some form of legalized introduction, then, the good or evil effects of competition, become questions meet for discussion by those who hope and pray for a higher standard.

The idea that dental colleges are not, as a rule, as thorough in their courses of study and discipline as they should be, was, in my opinion, originated and propagated mainly by the colleges themselves, through the ordinary methods of unprofessional competition. The managers of the various colleges are *all* honorable men and would not stoop to any act of discourtesy toward a brother dentist; let almost any one of them, however, be interviewed on matters pertaining to his own, or a rival school, and you will make the unwelcome discovery, that the sweet science of human duty, of doing unto others as we would have them do unto us, is lost sight of, for the time being, in the effort to disparage and dispose of all rivals, by insinuation, or by direct statement of the many imperfections that exist in their facilities for the instructing of students; or perhaps it is the low-standard of proficiency required of their candidates for graduation, or the classes are either too large to manage properly or too small to justify much notice, and so on *ad nauseum;* while his own, or some other, if remotely situated, is the only institution that is altogether lovely, wherein there is no deception; no promise unfulfilled; no mere pretension to superiority without good reason; where *all* the facilities for obtaining the most complete preparation and knowledge in dental science is found, in a manner suggesting the very acme of perfection.

Such sentiments expressed continuously, although usually "between you and me," by men whose position lends a world of wisdom to their words, must necessarily affect the profession generally, with the idea that all, or nearly all our colleges are devoid of the most essential requisites to an honest performance of the plainest obligations. An idea so obtained through motive of jealous rivalry, surely does not agree with existing facts. If we send young men of the right stamp, of common sense, possessed not only of a fair English education, and an acquaintance with Latin and Greek, but also a careful reading in the studies taught, we would find that the system of preparation as already arranged in the schools, would be all but perfect.

As it is, although the annual announcements may give notice that a preliminary examination into the mental capacity and morals of the applicant shall be made before acceptance as a student, not even one is refused admittance by any dental college. This taking in of all who apply, by no means involves the practice of an indiscriminate granting of diplomas. To my own knowledge, as many as four young gentlemen at a cêrtain college last session, who sought permission to enter the graduating class, were refused; and of those who are permitted to undergo the real trials of the final examination, generally as many as ten per cent. fail utterly, or get through only by "the skin of their teeth." These figures, I think, will obtain as an fair average in every dental college in our country, whether its classes be large or small; for the different classes are mentally and physically about alike; and the different colleges are also about alike in the qualifications they require of the graduates, notwithstanding the assertion they habitually make to each other, "I am holier than thou."

But in the light of the criticism we so often make against our system of dental education, it is not impertinent to ask, wherein lies the deficiency? Is it the quality or quantity of the branches of study and of practical work presented to the class? All you who have diplomas (from whatever school,) will agree with me that the fundimental branches, including anatomy, physiology, pathology, and chemistry, are fully, and in the main, ably presented, and all with especial reference to the needs of our profession; and in connection with the regular lectures on these subjects, work by the students in practical anatomy and in analytical chemistry, will be henceforth an absolute requirement. As for dental therapeutics and materia medica, it is not too much to say, that those who have within the last few years become full-fledged doctors of dental surgery, can formulate effective prescriptions with as much thoughtful elegance, as the most fastidious pharmacist could desire.

And what of the clinical department? A visit to an open infirmary will convince anyone that there need be no longer any lack of opportunity to furnish the students with abundance of practical employment in all the most interesting operations; "The poor,' says Boerhave, "are always with us." And when once made aware of the fact that they will be treated properly, they will thankfully take advantage of the gratuitous services as thankfully offered by all our colleges.

In regard to the duration of the session, it is perhaps just as well that there is a decided difference of opinion among those in authority; my own humble impression is, that a regular session of about five months in a dental college is as long as our students can be held down to interested and therefore profitable labor, and that such length of time is equal in point of systematic preparation, to eight or nine months in a medical college; for, aside from the fundamental branches, the dental student does not have to wade through the interminable swamps of medical theory and practice in order to find the blessed islets, where the flowers of truth are forever blooming; nor to search through the modified Pandora's box of materia medica and therapeutics, for the jewels of greatest value. His field is as a cultivated farm in the midst of a vast wilderness of uncertainty, and as the seasons come and go, he can gather with comparative ease, the golden grain from the places where judicious experience has sown the precious seed.

Nevertheless, should the aggregated wisdom of the profession decide that a new departure of whatever kind, whether in education or in practice, is necessary to our accepted or expectant progress, the direction will ever be, as in the past, toward the goal where lives the perfect realization of the heavenly maxim, "the greatest good to the greatest number."

And now, gentlemen, permit me to say in conclusion, that whatever of pleasure and benefit we may receive by meeting thus, at each recurring anniversary of the organization of our society, much of the credit is due to the happy forethought of our charter members, in selecting the early days of summer.

It is now, above all other periods of the year, that the tired worker, granted a brief respite from the toil inevitable to earnest and faithful performance of duty, can most fully realize the glorious loveliness of earth; the restful peace of mind and body, conferred by smiling nature, more soothing than balm of Gilead; and as we sped hence, thro' the most favored portion of our grand old Commonwealth, by hill and valley, which from the beginning of creation were, and shall ever be, so long as Time continues, fanned by archangels' wings, it seemed to me, that to be permitted to enjoy in all our senses, the arcadian sweetness of the season, the fruitful fields, the magnificent forests, the limpid streams, the birds, the blossoms of May commingling in the lap of June, is of far greater benefit, for the time being, to our physical and moral good, than all the wisdom we could obtain at the feet of Gamaliel; or than ancient votary could ever dream of finding as he kissed and quaffed in golden draught the grand elixer of the Alchemists.

